# Lipoteichoic Acid from *Staphylococcus aureus* Induces Lung Endothelial Cell Barrier Dysfunction: Role of Reactive Oxygen and Nitrogen Species

**DOI:** 10.1371/journal.pone.0049209

**Published:** 2012-11-15

**Authors:** Amy Barton Pai, Heena Patel, Alexander J. Prokopienko, Hiba Alsaffar, Nancy Gertzberg, Paul Neumann, Anjoli Punjabi, Arnold Johnson

**Affiliations:** 1 Department of Pharmacy Practice, Albany College of Pharmacy and Health Sciences, Albany, New York, United States of America; 2 Department of Pharmaceutical Sciences, Albany College of Pharmacy and Health Sciences, Albany, New York, United States of America; University of Florida, United States of America

## Abstract

Tunneled central venous catheters (TCVCs) are used for dialysis access in 82% of new hemodialysis patients and are rapidly colonized with Gram-positive organism (e.g. *Staphylococcus aureus*) biofilm, a source of recurrent infections and chronic inflammation. Lipoteichoic acid (LTA), a cell wall ribitol polymer from Gram-positive organisms, mediates inflammation through the Toll-like receptor 2 (TLR2). The effect of LTA on lung endothelial permeability is not known. We tested the hypothesis that LTA from *Staphylococcus aureus* induces alterations in the permeability of pulmonary microvessel endothelial monolayers (PMEM) that result from activation of TLR2 and are mediated by reactive oxygen/nitrogen species (RONS). The permeability of PMEM was assessed by the clearance rate of Evans blue-labeled albumin, the activation of the TLR2 pathway was assessed by Western blot, and the generation of RONS was measured by the fluorescence of oxidized dihydroethidium and a dichlorofluorescein derivative. Treatment with LTA or the TLR2 agonist Pam_(3)_CSK_(4)_ induced significant increases in albumin permeability, IκBα phosphorylation, IRAK1 degradation, RONS generation, and endothelial nitric oxide synthase (eNOS) activation (as measured by the p-eNOS^ser1177^:p-eNOS^thr495^ ratio). The effects on permeability and RONS were effectively prevented by co-administration of the superoxide scavenger Tiron, the peroxynitrite scavenger Urate, or the eNOS inhibitor L-NAME and these effects as well as eNOS activation were reduced or prevented by pretreatment with an IRAK1/4 inhibitor. The results indicate that the activation of TLR2 and the generation of ROS/RNS mediates LTA-induced barrier dysfunction in PMEM.

## Introduction

Sepsis is the second leading cause of death among patients with end-stage renal disease (ESRD) on hemodialysis (HD) [1]. Gram-positive organisms (e.g., *Staphylococcus spp*.) account for more than 70% of bacteremia/sepsis episodes primarily due to vascular access-related sources (e.g., tunneled central venous catheters) [1]. HD patients have a greater 30-day mortality risk compared to other populations with non-hospital acquired methicillin-resistant *Staphylococcus spp.* bacteremia [2].

Lipopolysaccharide (LPS) derived from the cell wall of Gram-negative bacteria is a known inducer of sepsis, the systemic inflammatory response syndrome (SIRS) and acute lung injury [3]. However, the cell wall components of pathogenic Gram-positive bacteria (e.g., *Staphylococcus aureus*) also mediate, at least in part, the vascular injury associated with the SIRS and acute lung injury [4], [5]. Pulmonary edema associated with increased vascular permeability is a complication of Gram-positive sepsis [6]. Principal components of the Gram-positive bacteria cell wall are lipoteichoic acid (LTA) [4], [5], [6] and peptidoglycan. LTA, unique to Gram-positive bacteria, consists of a hydrophilic region of polymers of ribitol phosphate or glycerophosphate substituted in the C2 position of glycerol by sugars in glycosidic linkage and D-alanine in an ester linkage [7]. Peptidoglycan is a transmembrane polymer consisting of glycan strands of N-acetylglucosamine and N-acetylmuramic acid that are, in Gram-positive bacteria, cross-linked by pentaglycine bridges. LTA derived from *Staphylococcus aureus* induces endothelial dysfunction, circulatory shock and organ injury in different models of Gram-positive sepsis [8], [9]. Peptidoglycan may act to amplify LTA-induced induce systemic inflammatory response syndrome [9], [10].

Classically, LTA binds to the toll-like receptor 2 (TLR2) which results in the activation of a MyD88-dependent pathway. In response to LTA, two pairs of intracellular adapter proteins–MyD88 and Toll-IL-1 resistance (TIR) domain-containing adapter protein (TIRAP) are recruited to the TLR receptor. Recruitment and activation of the IRAK proteins (1 and 4), follows and TRAF 6 subsequently activates the IKK complex (α,β and NEMO) which is the event immediately preceding NFκB activation and cytokine transcription [11], [12]. Peptidoglycan is a co-stimulator of the innate immune system in the presence of LTA, however, studies of highly purified peptidoglycan in the absence of LTA fail to stimulate TLR2 [13], [14]. Other pathways involved with LTA-TLR2 mediated alterations in endothelial function are not clear. It is known that reactive oxygen and nitrogen species mediate, in part, endothelial dysfunction during sepsis [15]. The literature indicates oxidant stress (e.g. reactive nitrogen and oxygen species) causes phosphorylation and redistribution of occludin and decreased levels of VE-cadherin associated with loss of peripheral cortical actin and increased actin stress fibers. The loss of actin peripheral bands is associated with disruption of α,β,γ catenins and actin binding proteins (e.g.,filamin) with increased cell-cell gap formation [16].

Yet, the ability of LTA to cause lung endothelial barrier dysfunction that is dependent on reactive oxygen/nitrogen species is not known; therefore, the aim of this study is to investigate the mechanism of LTA –induced endothelial barrier dysfunction in a lung microvessel endothelial cell monolayer model.

**Figure 1:**
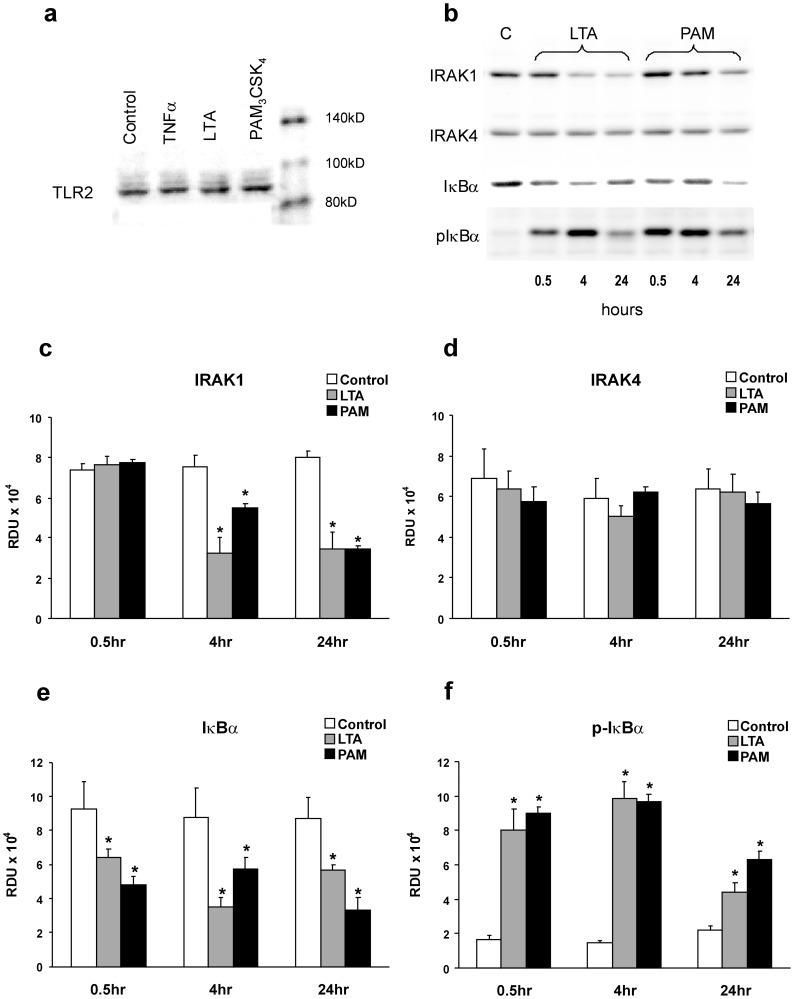
LTA- or PAM-induced activation of the TLR2 pathway in pulmonary microvessel endothelial monolayers (PMEM). (*a*) Representative Western blot of TLR2 in PMEM after treatment with vehicle, TNFα 100 ng/mL, LTA from *S. aureus* and PAM (both 30 µg/mL) for 1 hour, confirming presence of receptors. (*b*) Representative Western blots of IRAK1, IRAK4, IκBα, and p-IκBα^Ser32/36^ from Control (C) or 0.5, 4 and 24 hour LTA or PAM-treated (both 10 µg/mL) PMEM and (*c–f*) the blot band densities in Relative Density Units (RDU) for these proteins from all blots of similar treatments. Values represent means ± SEM (N ≥4). * p<0.05 vs. Control.

## Materials and Methods

### Reagents

All reagents were obtained from Sigma Chemical Company (St. Louis, MO) unless otherwise noted.

### Cells

#### Pulmonary microvessel endothelial cell culture

Rat lung microvessel endothelial cells (RLMVEC) were studied using our previously published methods [17]. In brief RLMVEC were obtained at 4th passage (Vec Technologies, Rensselaer, NY). The preparations were identified by Vec Technologies as pure populations by *1*) the characteristic “cobblestone” appearance as assessed by phase contrast microscopy, *2*) the presence of factor VIII-related antigen (indirect immunofluorescence), *3*) the uptake of acylated low-density lipoproteins, and *4*) the absence of smooth muscle actin (indirect immunofluorescence). For all studies, RLMVEC were cultured from 4 to 10 passages in culture medium, MCDB-131 complete media (VEC Technologies) supplemented with 20% fetal bovine serum (FBS) (Hyclone; Hyclone Laboratories, Logan, UT). The cells were maintained in 5% CO_2_ plus humidified air at 37°C. A confluent pulmonary microvessel endothelial cell monolayer (PMEM) was reached within two to three population doublings, which took 3–4 days.

**Figure 2:**
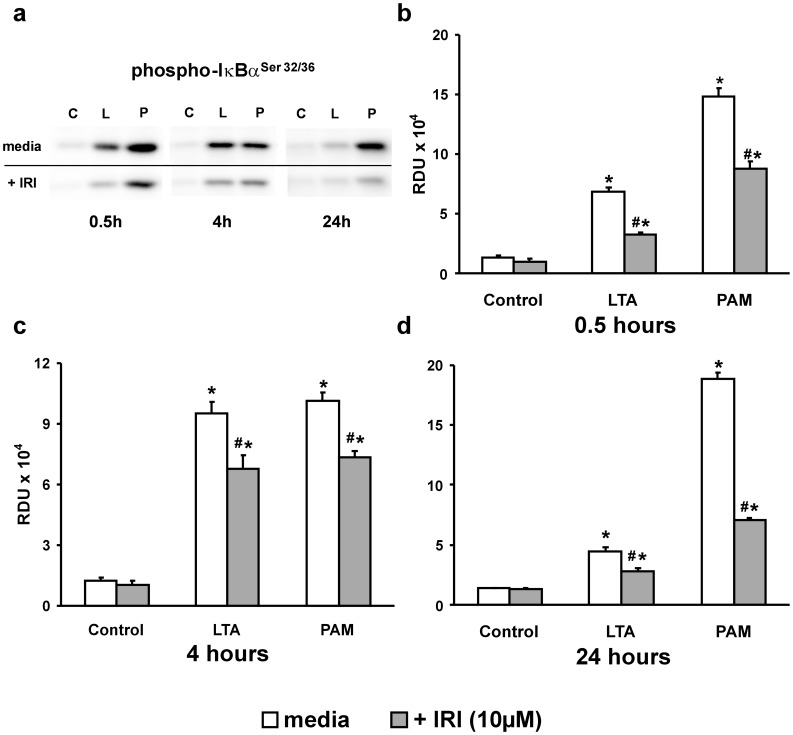
IRAK1/4 inhibition of the TLR2 pathway. (*a*) Western blots of p-IκBα^Ser32/36^ from 0.5, 4 and 24 hour Control (C), LTA (L) or PAM (P) -treated (both 10 µg/mL) PMEM in the absence (upper bands) or presence (lower bands) of IRAK1/4 inhibitor (IRI: 10 µM). (*c–d*) Western blot band densities in Relative Density Units (RDU) for p-IκBα^Ser32/36^ from all blots represented by (a). Values represent means ± SEM (N ≥4). * p<0.003 vs. Control, **#** p<0.02 vs. TLR2 agonist alone.

### Treatments

#### TLR2 agonists/inhibitors

Purified LTA (InvivoGen, San Diego, CA) from *Staphylococcus aureus* in a stock solution of 2 mg/ml was used (endotoxin <1.25 EU/mg). This preparation of LTA from *S. aureus* results in 10 times less endotoxin according to the gel clot LAL Assay than the standard preparation. It highly activates TLR2 and no other TLRs including TLR4. PMEM were treated with LTA at 10–30 µg/ml, because preliminary dose-response studies indicate these doses consistently induce a permeability increase. This range of LTA doses are biologically relevant concentrations that can be achieved in the dead space of an intravascular catheter with biofilm (10^7^ to 10^9^ CFU) present [18], [19]. In addition to LTA, PMEM were treated with the synthetic bacterial lipoprotein and TLR2-TLR1 ligand positive control, N-Palmitoyl-S-[2,3-bis(palmitoyloxy)-(2RS)-propyl]-[R]-cysteinyl-[S]-seryl-[S]-lysyl-[S]-lysyl-[S]-lysyl-[S]-lysine (PAM_(3)_CSK_(4)_ [PAM]) (InvivoGen), at 10–30 µg/ml [20]. The interleukin receptor-associated kinases 1 and 4 (IRAK1/4) inhibitor (IRI), N-(2-Morpholinylethyl)-2-(3-nitrobenzoylamido)-benzimidazole [21] (EMD Chemicals, Gibbstown, NJ) was used to block the TLR2 signaling cascade. PMEM were pretreated for 2 hours with IRI alone (10 µM) prior to co-treatment with IRI and TLR2 agonists.

**Figure 3:**
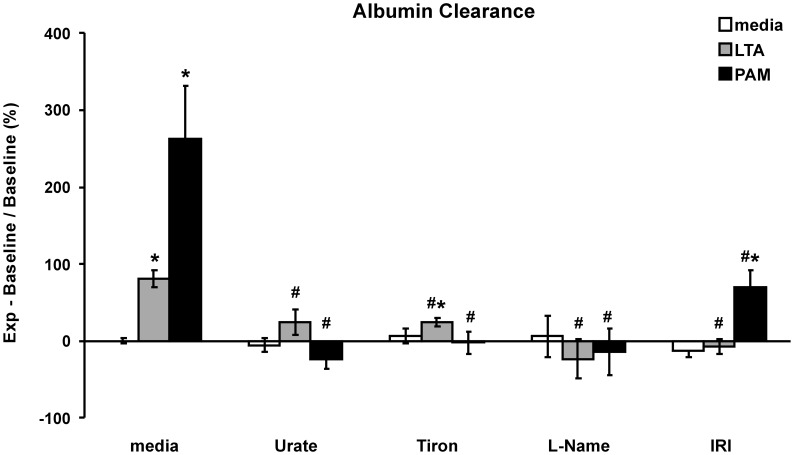
TLR2 agonist-induced PMEM barrier dysfunction. The PMEM clearance rate over 1 hour of Evans Blue-labeled albumin was measured in a transwell system. Cell monolayers were treated for 24 hours with LTA (30 µg/mL) or PAM (10 µg/mL) alone or co-treated with RNS scavenger, Urate (5 µM), ROS scavenger, Tiron (5 mM), or NOS inhibitor, L-NAME (100 µM) or co-treated for 24 hours following a 2 hour pretreatment with IRAK1/4 inhibitor, (IRI: 10 µM). Units are % change in µL/min from Control values (media alone). Values represent means ± SEM (N ≥4). * p<0.02 vs. media alone, **#** p<0.02 vs. TLR2 agonist alone.

#### Anti-ROS/RNS/eNOS agents

Uric Acid (Urate) (5 µM) was used to inhibit peroxynitrite(ONOO^–^), 4,5 Dihydroxy-1,3-Benzene-Disulfonic Acid (Tiron) (5 mM) was used to scavenge •O_2_- and L-nitroso arginine methyl ester (L-NAME; 100 µM) was used to inhibit eNOS. We have previously shown that Urate and Tiron scavenge TNF-induced ONOO^–^ and •O_2_^−^ [17], [22], [23]. We previously showed that L-NAME inhibits nitric oxide generation as a competitive antagonist of L-arginine [23]. Finally, we have previously shown that Urate, Tiron and L-NAME have no effect on endothelial cell viability [12], [16], [17]. RLMVEC monolayers were treated with Vehicle, Urate, Tiron or L-NAME alone or co-treated with LTA or PAM. Trypan blue exclusion studies showed average viability of controls, LTA and PAM treatments of 96.5%, 98.2%, 96.2% (0.5 hour), 96.7%, 98.0%, 93.0% (4 hours), and 94.7%, 92.7% 93.0% (24 hours), respectively.

**Figure 4:**
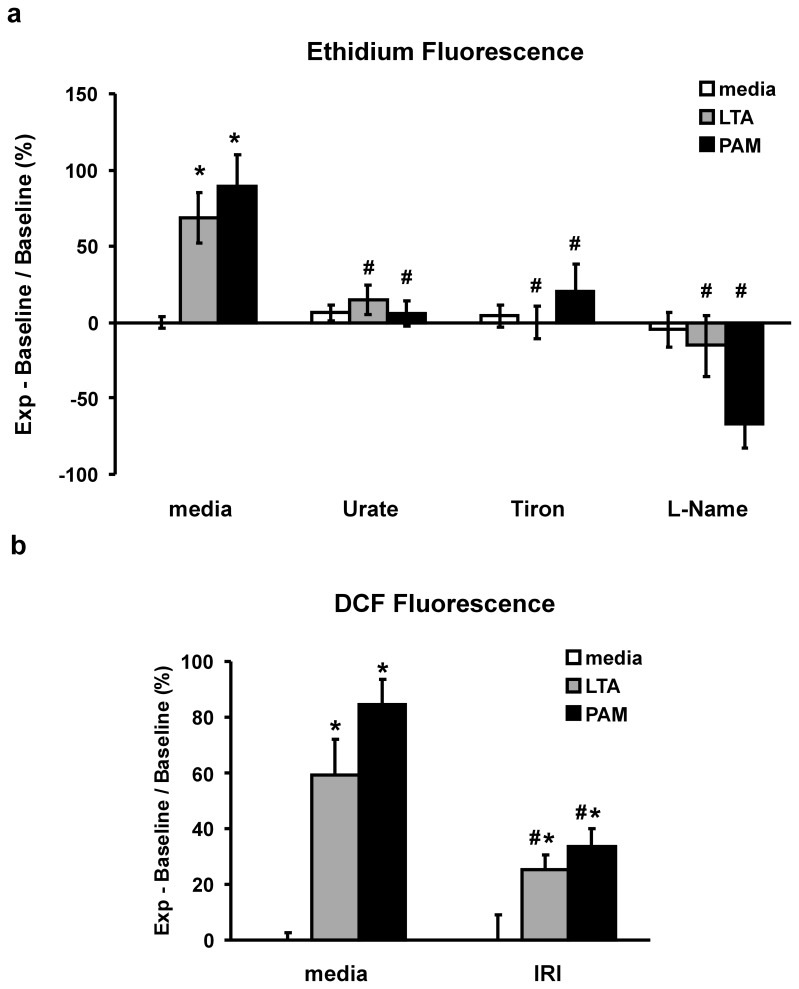
TLR2 agonist-induced production of reactive oxygen/nitrogen species (RONS). (*a*) Fluorescence of oxidized dihydroethidium (DHE) in cell sonicate after a 30 minute treatment of PMEM with LTA (30 µg/mL) or PAM (10 µg/mL) alone or co-treated with RNS scavenger, Urate (5 µM), ROS scavenger, Tiron (5 mM), or NOS inhibitor, L-NAME (100 µM). (*b*) Fluorescence of oxidized DCFDA in intact cell monolayers in 96-well plate following a 2 hour treatment with LTA or PAM alone (both 10 µg/mL) or co-treatment following a 2 hour pretreatment with IRAK1/4 inhibitor, (IRI: 10 µM). Units are % change in relative fluorescence units from Control values (media alone). Values represent means ± SEM (N ≥4). * p<0.01 vs. media alone, **#** p<0.04 vs. TLR2 agonist alone.

#### Treatment medium

For all studies, incubation of PMEM with LTA, PAM, Urate, Tiron, L-NAME, IRI and all corresponding controls were performed with phenol red-free DMEM (pf-DMEM) (Life Technologies, Grand Island, NY) supplemented with 10% FBS (20% FBS for 24 hour studies) to avoid a potential antioxidant effect of phenol.

**Figure 5:**
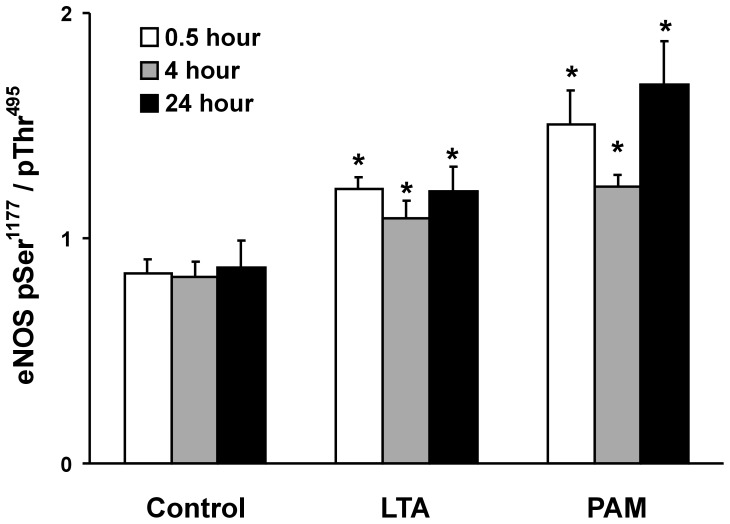
TLR2 agonist-induced increase in endothelial nitric oxide synthase (eNOS) activity. An index of eNOS activity was calculated from the ratio of Western blot band densities of eNOS activation site phosphorylation over eNOS inhibitory site phosphorylation (phospho-eNOS (Ser1177)/phospho-eNOS (Thr495)). PMEM were treated for 0.5, 4 and 24 hours with LTA or PAM (both 10 µg/mL). Values represent means ± SEM (N ≥4). * p<0.05 vs. Control (media alone).

**Figure 6:**
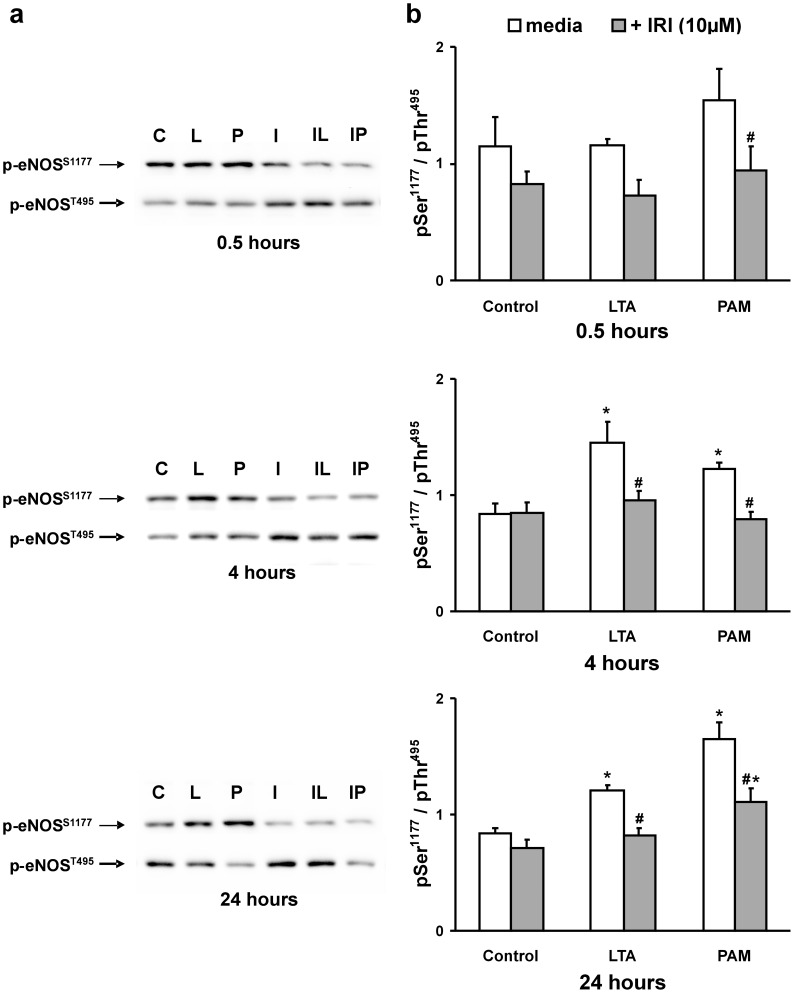
TLR2 agonist-induced IRAK activity increases endothelial nitric oxide synthase (eNOS) activity. PMEM pre-treated for 2 hours in the absence or presence of IRAK1/4 inhibitor (IRI: 10 µM) were then co-treated with LTA or PAM (both 10 µg/mL) for 0.5, 4, and 24 hours. (*a*) Representative Western blots for each treatment period of the eNOS activation site phosphorylation (p-eNOS^S1177^, upper band) and the eNOS inhibitory site phosphorylation (p-eNOS^T495^, lower band). The labels are: Control (C), LTA (L), PAM (P), IRI alone (I), IRI+LTA (IL), and IRI+PAM (IP). (*b*) Western blot band density ratios of p-eNOS^S1177^/p-eNOS^T495^ for all blots of all treatment groups at each time point represented in panel (a). Values represent means ± SEM (N ≥4). * p<0.03 vs. media alone, **#** p<0.03 vs. TLR2 agonist alone.

**Figure 7:**
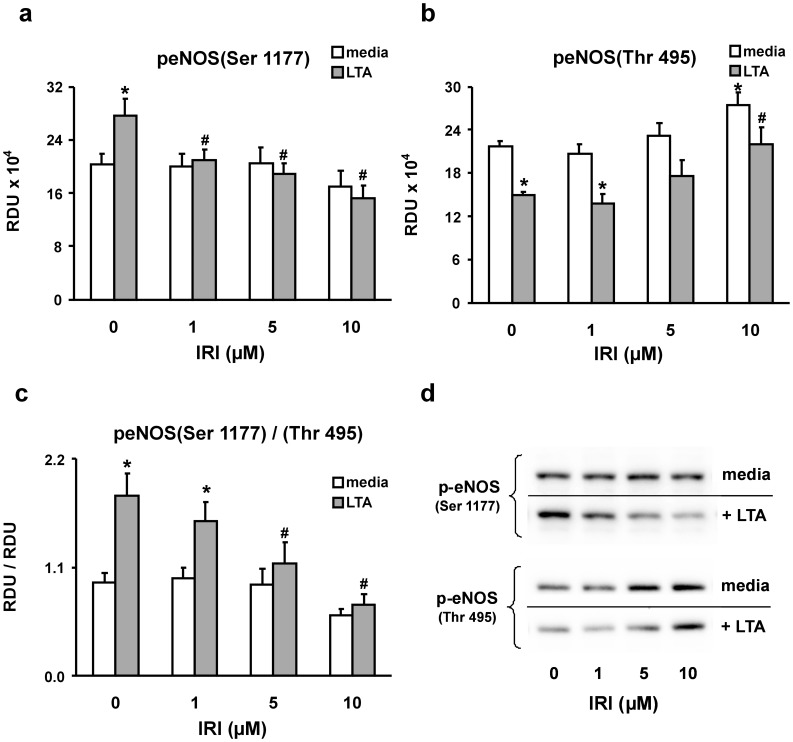
IRAK mediates eNOS activation and inhibitory site phosphorylation in LTA-treated PMEM. Western blots were generated from PMEM pre-treated for 2 hours in the absence or presence of increasing concentrations of IRAK1/4 inhibitor (IRI) and then co-treated with LTA (10 µg/mL) for 4 hours. (*a*) The Western blot band densities of phosphorylated serine 1177 of eNOS, its major activation site. (*b*) The Western blot band densities of phosphorylated threonine 495 of eNOS, a major inhibitory site. (*c*) The ratios of the band densities in panel (a) over those in panel (b). The units for panels a – c are relative density units (RDU). (*d*) Representative Western blots. Values represent means ± SEM (N ≥4). * p<0.03 vs. media alone, **#** p<0.03 vs. LTA alone.

### Endothelial Permeability *Assay*

The assay of endothelial cell monolayer permeability is adapted from our previously described technique [17]. Transwells (6.5 mm diameter, 8 µm pore size; Corning Costar, Corning, NY) were coated with Rat Tail Collagen Type 1 (BD Biosciences, Bedford, MA). RLMVEC (0.1×10^6^) in MCDB-131 were plated in the Transwells according to manufacturer’s instructions and allowed to reach confluence within 3–5 days (37°C, 5% CO_2_). The experimental apparatus for the study of transendothelial transport in the absence of hydrostatic and oncotic pressure gradients have been described [17]. In brief, the system consists of two compartments separated by a microporous polycarbonate membrane lined with the endothelial cell monolayer as described above. The luminal (upper) compartment (0.1 ml) was suspended in the abluminal (lower) compartment (0.6 ml). The entire system was kept in a CO2 incubator at a constant temperature of 37°C. The fluid height in both compartments was the same to eliminate convective flux.

Endothelial permeability was characterized by the clearance rate of Evans Blue-labeled albumin [24]. A buffer solution containing Hanks’ balanced salt solution (Life Technologies) containing 20mM HEPES buffer and 0.5% bovine serum albumin (BSA) was used on both sides of the monolayer (HHB buffer). The luminal compartment buffer was labeled with a final concentration of 0.057% Evans blue dye in a volume of 100 µl. The absorbance of free Evans blue in the luminal and abluminal compartments was always <1% of the total absorbance of Evans blue in the buffer. At the beginning of each study a luminal compartment sample was diluted 1∶100 to determine the initial absorbance of that compartment. Abluminal compartment samples (100 µl) were taken every 10 min for 60 min. To maintain a constant hydrostatic pressure, the sample removed from the abluminal compartment was replaced with an equal volume (100 µl) of HHB buffer. The absorbance of the samples was measured in a BioTek Synergy2 plate reader (BioTek Instruments, Winooski, VT) at 620 nm. The clearance rate of Evans blue-labeled albumin was determined by least-squares linear regression between 10 and 60 min for the control and experimental groups. Mean ± SEM albumin permeability for control experiments was 0.035±0.004 µL/min.

### Immunoblot Analysis

#### Preparation of PMEM lysates

RLMVEC were seeded into 6 or 12-well plastic culture plates and incubated for 3–4 days until confluent. After interventions, the PMEM were washed on ice two times with ice-cold PBS, then ice-cold extraction buffer (Tris HCl: 10 mM-pH 7.5; SDS: 0.1%; Triton X-100∶0.5%; Sodium Deoxycholate: 0.5%; DTT: 0.1 mM) supplemented with 1x mammalian protease inhibitor cocktail and 1x phosphatase inhibitor cocktails 1 and 2 was added to the cells. Lysates were cleared by centrifugation at 18,500×g for 45 minutes at 4°C. Cell lysate protein concentrations were determined by BCA assay (Thermo Scientific, Rockford, IL) against BSA protein standards. All samples were normalized for protein content, diluted 4∶1 in 5x Laemmli buffer, heated 10 min at 95°C, and stored at −80°C.

#### Western blot

The lysate proteins were separated by SDS-PAGE on 9%, 1.5 mm thick, 15-lane Mini-Protean III gels using standard procedures (Bio-Rad, Hercules, CA). All lanes were loaded so that each lane contained 16 µg of total protein. The polyacrylamide gels were transferred to polyvinylidene difluoride (PVDF) membranes (Immobilon-P; Millipore, Bedford, MA) at 125 volts for 1 hour with Towbin’s transfer buffer. The PVDF membranes were then blocked with blotto (5% nonfat dry milk in TTBS [Tween 20∶0.05%; Tris HCl: 10 mM, pH 7.5; NaCl: 100 mM]) for 30 minutes at room temperature (RT).

#### Immunoprobing

TLR2 was identified with rabbit polyclonal anti-TLR2 (LS-C819, LifeSpan Biosciences, Seattle, WA) and interleukin receptor-associated kinases 1 and 4 were detected with rabbit polyclonal anti-IRAK1 (sc-7883, Santa Cruz Biotechnology, Santa Cruz, CA) and anti-IRAK4 (Cell Signaling Technology, Danvers, MA). The metabolic state of IκBα was visualized with mouse monoclonal anti-IκBα and anti-phospho-IκBα (Ser32/36) and that of eNOS with rabbit polyclonal anti-phospho-eNOS (Ser1177) and anti-phospho-eNOS (Thr495) (Cell Signaling,#’s 4814, 9246, 9571, 9574, respectively). Blots were incubated in these primary antibodies at a 1∶2000 dilution in either blotto or TTBS with 5% BSA, according to manufacturers instructions, overnight at 4°C.

Secondary antibody blot incubation was 1–1.5 hours at RT in blotto with donkey anti-rabbit or goat anti-mouse IgG-HRP conjugates (Santa Cruz) diluted 1∶5000. Goat anti-biotin-HRP, 1∶5000, was included in the secondary antibody incubation to detect biotin-labeled molecular weight markers (Cell Signaling) on the blots. Unbound material was removed from the blots when required by washing five times for 5 min each with TTBS at RT. Signal was generated with a 1∶1 dilution of SuperSignal West Dura- and SuperSignal West Pico- Chemiluminescent Substrates (Thermo Scientific).

Blots were stripped prior to reprobing with Restore PLUS Western Blot Stripping Buffer (Thermo) for 15 minutes at RT.

#### Detection and analysis

Blots were imaged with a Chemidoc XRS (Bio-Rad) and net band intensity units were measured with Image Lab image analysis software (Bio-Rad). All blots contained equal numbers of samples of each experimental treatment. The mean band intensity of all samples on an individual blot was normalized across all blots to an arbitrarily chosen value to compensate for interblot variability and individual sample band values were then adjusted proportionally to reflect that normalized mean.

### Fluorescence Detection of RONS in PMEM

#### Ethidium

RLMVEC (0.3×10^6^/2.0 ml of culture medium) were plated in six-well culture dishes and allowed to grow to confluence. After treatment, PMEM were washed 1X with PBS- and incubated with 1 µM dihydroethidium (DHE) (Life Technologies) in phenol free DMEM with 10% FBS (1 ml/well) at 37°C for 0.5 h. PMEM were then washed on ice 2x with ice-cold DPBS- to remove free probe and scraped with 1 ml of ice-cold DPBS- into microtubes. The cells were centrifuged at 8,000 *g* for 10 min at 4°C, and the pellet was resuspended in 0.4 ml of ice-cold DPBS. The cell suspensions were sonicated on ice for 15 seconds, and 100 µl/well sonicate were added to Falcon 96-well black microplates (Becton Dickinson, Franklin Lakes, NJ) in quadruplicate. Probe-free cell sonicate was used for blanks.

#### Fluorescein

RLMVEC (2.5×10^4^/200 µl of culture medium) were plated in Costar 96-well black clear-bottom culture plates (Corning, Corning, NY, #3603) and grown to confluence. After treatment, PMEM were washed 1X with HBSS (Fisher Scientific, Waltham, MA) and incubated with 10 µM 6-Carboxy-2′,7′-Dichlorodihydrofluorescein Diacetate, Di(Acetoxymethyl Ester) (DCFDA) (Life Technologies) in HBSS (75 µl/well) at 37°C for 30 minutes. DCFDA probe was then removed and replaced with 50µl/well HBSS prior to fluorescence measurement.

Fluorescence was measured with a BioTek Synergy2 plate reader (BioTek Instruments) using excitation and emission wavelengths of 485 nm and 620 nm (DHE) or 485 and 528 nm (DCFDA), respectively. Fluorescence was presented as percentage change from control by the formula [(F*t*exp- F*t*control)/F*t*control]×100%, where F*t*exp  =  fluorescence at any time after treatment in a given sample and F*t*control  =  fluorescence of the respective untreated control group.

### Statistics

All data points are mean values of treatment samples. All samples were generated over 2 to 4 experiments within which there were 2 to 4 replicates for each treatment, except for 96-well microplate format studies, which contained 8–12 replicates per treatment. Each PMEM well represents a single sample. There are at least 4–10 samples per group in all studies. A one-way analysis of variance was used to compare values among the treatments. If significance among treatments was noted, a post hoc multiple-comparison test was done with a Bonferroni (parametric-equal variance) or a Duncan (nonparametric-unequal variance) test to determine significant differences among the groups. All data are reported as means ± SE. Significance was at *P*<0.05.

## Results

### LTA Induces Activation of the TLR-2 Pathway in Pulmonary Endothelium

The presence of TLR2 in RLMVEC was confirmed by detection on Western blots with anti-TLR2. TLR2 was identified on the pulmonary endothelial cells independent of 1 hour challenge with vehicle, TNFα, LTA, or PAM. A representative Western blot is shown in Figure 1a. Known consequences of TLR pathway activation, primarily shown in leukocytes, include IRAK1 activation, phosphorylation, and depletion as well as phosphorylation and depletion of IκBα during NFκB activation [25]. Representative Western blots of IRAK1, IRAK4, IκBα and phospho-IκBα-Ser^32/36^ (p-IκBα) from PMEM treated with either LTA or PAM for 0.5, 4 and 24 hours compared to vehicle are shown in Figure 1b and the band density data for all similar blots is given in Figure 1c–f. Both LTA and PAM caused a late (4–24 hr.) sustained decrease in IRAK1 with no changes in IRAK4, and a rapid (0.5 hr.) and sustained (4–24 hr.) increase in p-IκBα with a concomitant decrease in the total IκBα cellular pool. These results suggest activation of the Toll-like receptor pathway; NFκB activation and IRAK1 degradation subsequent to its activation through TLR signaling.

To verify these results, an IRAK1/4 inhibitor (IRI) was utilized to block the downstream effects of TLR2 activation. An initial PMEM dose response to IRI was performed that indicated a 2 hour pretreatment with 10 µM IRI was optimal for curtailing IκBα phosphorylation following LTA treatment while still maintaining monolayer integrity. Higher doses of IRI (25–50 µM) resulted in large gaps in the cell monolayer visible under low resolution phase microscopy (data not shown). Representative Western blots of cell lysates from PMEM treated with either LTA or PAM in the presence of IRI (10 µM) for 0.5, 4 and 24 hours (Figure 2a) reveal a marked decrease in the generation of p-IκBα. The p-IκBα band density data from all blots of this treatment group (Figure 2b–d) show that this decrease is significant and sustained throughout the study period (∼28%–62% less than agonist alone, p≤0.02, for all treatments). The data of Figures 1 and 2 support the idea that both LTA and PAM activate TLR2 in rat lung microvessel endothelial cell monolayers and that this activation can be at least partially inhibited with an IRAK1/4 inhibitor.

### Activation of the TLR-2 Pathway Induces Endothelial Barrier Dysfunction Mediated by Reactive Oxygen/nitrogen Species

Endothelial cell monolayer permeability changes were assayed 24 hours after treatment with either LTA or PAM. Both LTA and PAM significantly increase permeability to albumin compared to control PMEM (mean ± SEM: 81±11% and 262±70% increase versus untreated controls, p>0.01, respectively). This increase in permeability is ameliorated or prevented by co-treatment with the superoxide scavenger tiron, the reactive nitrogen species scavenger, urate, the NOS inhibitor L-NAME, or by treatment with the IRAK1/4 inhibitor IRI (Figure 3).

There is a marked increase in DHE oxidation after a 30 minute treatment of PMEM with either LTA or PAM,(mean ± SEM: 69±17% and 89±20% increase versus untreated controls, p>0.01, respectively). Co-treatment of LTA- or PAM-treated monolayers with tiron, urate or L-NAME prevents the oxidation of DHE (Figure 4a). Treatment of PMEM grown in 96-well plates with LTA or PAM for 2 hours resulted in a significant increase in DCFDA oxidation whereas the degree of DCFDA oxidation in LTA- or PAM-treated PMEM was significantly reduced in the presence of IRI (mean ± SEM: 25.2±5.2% *vs* 59.3±13%, LTA+IRI *vs* LTA alone, p<0.04 and 33.4±6.7% *vs* 84.2±9.6%, PAM+IRI *vs* PAM alone, p<0.01) (Figure 4b).

### Role of RNS: eNOS Activation

Treatment of RLMVEC monolayers with either LTA or PAM is associated with DHE oxidation that is inhibited by urate or L-NAME, suggesting a role for reactive nitrogen species; thus, eNOS was examined. Phosphorylation of eNOS at ser^1177^ activates eNOS; conversely, phosphorylation of eNOS at thr^495^ is associated with a decrease in eNOS activity [26]. A measure of eNOS activity was therefore determined by the ratio of phospho-eNOS-ser^1177^/phospho-eNOS-thr^495^ (pSer^1177^/pThr^495^). Both LTA and PAM increased the ratio of pSer^1177^/pThr^495^, suggesting eNOS activation in response to LTA or PAM. This effect was early (0.5 hours) and sustained over 24 hours (Figure 5). In the presence of IRI, the activation of eNOS in response to LTA or PAM treatment was virtually eliminated. Representative Western blots of p-eNOS-ser^1177^ and the corresponding p-eNOS-thr^495^ bands from PMEM treated with LTA or PAM in the absence or presence of IRI for 0.5, 4, and 24 hours are shown in Figure 6a. The cumulative eNOS pSer^1177^/pThr^495^ band density ratios for these treatments reveal that eNOS activity in PMEM, as defined herein, remains at control levels after exposure to LTA or PAM when an IRAK1/4 inhibitor (IRI) is present. Figure 7 further illustrates the dynamics of eNOS activation and inhibitory site phosphorylation in PMEM exposed to LTA for 4 hours in the presence of increasing amounts of IRI. In the absence of inhibitor, LTA induces both an increase in pSer^1177^ and decrease in pThr^495^ resulting in a large eNOS pSer^1177^/pThr^495^ ratio increase relative to control. The level of pSer^1177^ after LTA exposure rapidly falls as the concentration of IRI is increased whereas the level of pSer^1177^ in the absence of LTA remains relatively unaffected (Figure 7a). Conversely, both control and LTA-exposed PMEM levels of pThr^495^ rise with an increase in IRI concentration (Figure 7b). The LTA-induced decrease in pThr^495^ is not eliminated by IRI but contributes less to the overall eNOS pSer^1177^/pThr^495^ ratio change relative to the magnitude of the IRI-induced increase in pThr^495^ and decrease in pSer^1177^. This results in a dramatic fall in the pSer^1177^/pThr^495^ ratio (eNOS activity) of LTA-exposed PMEM with increasing IRI concentration (Figure 7c). Representative Western blots are shown in Figure 7d which illustrate this effect.

## Discussion

In the present study, LTA activated TLR2 dependent pathways because: (1) TLR2 is detected in the RLMVEC, (2) LTA caused specific depletion of IRAK1, and (3) LTA caused increased p-IκBα-ser^32/36^ associated with decreased IκBα. The synthetic, TLR1/2- specific agonist PAM also caused a depletion of IRAK1 and increased p-IκBα-ser^32/36^ similar to LTA which further supports the idea that TLR2 mediates the effect of LTA. Recognition of PAM is mediated by TLR2 cooperatively with TLR1 through their cytoplasmic domains to induce the signaling cascade leading to the activation of NFκB. While TLR1 cooperates in initiation of signaling by PAM, signaling is mediated via the TLR 2 pathway and is thus the receptor of significance in mediating the deleterious effects of both LTA and PAM. Importantly, the agonists have no TLR4 activity [27], [28]. Signaling activated by LTA is documented to be mediated via TLR2. PAM is a widely proven agonist of TLR2 and, although it causes dimerization of TLR2 with TLR1, in TLR2 knock-out studies, neither PAM nor LTA activate the Toll-receptor cascade [29], [30]. Finally, the activation of the TLR2 pathway was not a non-specific effect of endothelial cell activation because TNF did not affect IRAK1 despite the TNF induced increase in p-IκBα-ser^32/36^ (data not shown). Importantly, both LTA and PAM caused similar increased albumin clearance rates which supports the idea that TLR2 receptor activation mediates a permeability effect in the rat pulmonary microvessel endothelial cells. The ultrapure LTA from *S. aureus* used in our experiments contains minimal endotoxin; thus, these observed effects can confidently be attributed to LTA and TLR2 activation. However, it should be noted that there are other Gram-positive cell wall components that are TLR2 ligands that may contribute to endothelial activation such as a principal lipoprotein peptidoglycan. Peptidoglycan has been reported to be a more potent inducer of inflammation than LTA and to act synergistically with LTA [8], [29]. A recent study examined the effects of TLR2 agonists (e.g., peptidoglycan-associated lipoprotein and PAM) on human endothelial cell function [28]. The TLR2 agonists activated human umbilical vein, lung, and coronary artery endothelial cells, indicated by increased IL-6 and E-selectin production. In addition, increased FITC-labeled albumin permeability was demonstrated in human umbilical vein endothelial cell monolayers treated with 5 to 15 µg/mL of PAM for 24 hours. Xing et al. recently demonstrated that LTA doses ranging from 50 to 300 ng/mL induced significant barrier dysfunction in human pulmonary artery endothelial cells measured by transendothelial electrical resistance [9]. The authors also showed that intratracheal administration of LTA alone as well as in combination with peptidoglycan induced lung injury *in vivo* in a murine model that measured bronchial alveolar lavage cell count and protein concentration. LTA also induced activation of key intracellular signaling proteins, evidenced by increased phosphorylation of p38 MAP kinase, HSP 27 and at later time points ERK 1/2 MAP kinase; and similar to our findings, the authors observed degradation of IκBα.

Yet, to the best of our knowledge our study represents the first to show that activation of the TLR2 pathway by ultrapure LTA or PAM induces increased permeability by a mechanism mediated by ROS/RNS generation in lung endothelial cells. LTA and PAM induced significant increases in endothelial monolayer permeability and DHE oxidation which were prevented by the superoxide radical (•O_2_^−^) scavenger tiron, the peroxynitrite (ONOO−) scavenger urate, and the eNOS inhibitor L-NAME. This indicates LTA induces the generation of reactive oxygen species (i.e., scavenging by tiron) and reactive nitrogen species (i.e., scavenging by urate and eNOS inhibition with L-NAME) that increase endothelial permeability. The mechanism for the LTA induced oxidant dependent increase in permeability is not clear from the present study. However, the literature indicates oxidant stress causes phosphorylation and redistribution of occludin and decreased levels of VE-cadherin associated with loss of peripheral cortical actin and increased actin stress fibers. The loss of actin peripheral bands is associated with disruption of α,β,γ catenins and actin binding proteins (e.g.,filamin) with increased cell-cell gap formation. Moreover PLC, PLD and PLA2 are activated during oxidant stress resulting in increased endothelial kinase (e.g., PKC) activity and generation of lipid mediator (thromboxane A2) [16], [17]. Previous work by our group and others have shown that ROS/RNS mediate endothelial dysfunction [16]. TLR2 activation induces AKT activation and AKT activates eNOS via phosporylation of eNOS-ser-1177 [34]. Also, previous studies indicate that the TLR2 receptor agonist, LTA, induces endothelial dysfunction: therefore, we studied the relationship between TLR2 activation and ROS/RNS production. As an example of this relationship, in the present study, the TLR2 receptor agonists LTA and PAM both cause an increase in the activation of eNOS evidenced by the increase in phospho-eNOS-ser^1177^/phospho-eNOS-thr^495^ which results in increased production of ROS and RNS.

In macrophages, LTA exposure is associated with increased iNOS and NO release [35]. Both LTA and PAM induced eNOS activation, the likely source of NO and peroxynitrite via the interaction between NO and •O_2_^−^ → ONOO^−^. We previously showed during TNF-induced endothelial injury that the protective effect of L-NAME was due to competitive inhibition of the substrate L-arginine resulting in decreased nitric oxide, and that urate is a scavenger of ONOO- [22]. Although the limited specificity of urate as a peroxynitrite scavenger is appreciated, reduction of both endothelial cell monolayer permeability and RO/NS generation after either LTA or PAM co-treatment with urate, or with L-NAME, a specific NOS inhibitor, provides compelling evidence for a RNS mediated injury pathway. IRAK1/4 inhibition also prevented the LTA-induced increase in permeability as well as minimizing the LTA-induced oxidation of DCFDA. Inhibition of IRAK had a profound effect on phosphorylation patterns of the major effector sites of eNOS, essentially reversing the TLR2 agonist-induced changes and eliminating eNOS activation. It is known that eNOS is activated by the Akt dependent phosphorylation of eNOS-ser^1177^ whereas PKCδ has been shown to be responsible for phosphorylation of the eNOS-thr^495^ inhibitory site [36], [37]. TLR-dependent Akt activation occurs via a TLR/TIRAP/MyD88/PI3K/Akt pathway [38], [39] and Dunne et al found that both IRAK1 and IRAK4 directly phosphorylate TIRAP (Mal) [40]. This suggests a direct role for IRAK in the TLR signaling cascade leading to the activation of Akt. LTA increases PKCδ activity in fibroblasts [41] and Tiwari et al have reported direct interaction between IRAK1 and PKCδ in monocytes [42]. Additionally, Xiong et al demonstrated that NO inhibits IRAK activity in splenocytes, macrophage, and dendritic cells [43]. These findings are consistent with the noted TLR2 agonist-dependent, and IRAK inhibitor reversible, increase in the p-eNOS-Ser^1177^/p-eNOS-thr^495^ ratio of the present study. These findings along with our observations also potentially describe a previously unreported self-regulating relationship between the activities of IRAK and eNOS.

In the present study, the protective effect of Tiron indicates the generation of •O_2_^−^. NADPH-oxidase dependent •O_2_^−^ has been shown to mediate various models of endothelial injury such as TNF-induced increased endothelial permeability [44]. NADPH-oxidase consists of a NOX2 (gp91phox), p47, p22 and Rac regulated by multiple phosphorylation and translocation events. In smooth muscle cells, LTA induces MAPK activation mediated by a TLR2/MyD88/PI3K/Rac1/Akt pathway [45]. In bone marrow-derived macrophages, Maitra et al demonstrated that IRAK-1 associated with and activated small GTPase Rac1, a known activator of NOX-1 oxidase enzymatic activity [46]. The NOX 4 homolog is also expressed in endothelial cells in which its activity may be independent of Rac and p47phox. In human aortic endothelial cells exposed to LPS, Park et al. have demonstrated the critical role of NOX 4 derived ROS in LPS-induced monocyte adhesion and migration [47]. Recently Lee et al. have shown in smooth muscle cells that treatment with LTA 50 µg/mL induced ROS generation that was inhibited by the NOX inhibitors DPI and apocynin, suggesting ROS generation is NOX dependent. The authors demonstrated NADPH-oxidase activation via a TLR2/MyD88/TRAF6/c-Src/p47phox complex [48]. Indeed, it has been shown that the Src family of tyrosine kinases activates the NADPH-oxidase complex [49]. Finally, the un-coupling of eNOS because of modification of the protein by reactive oxygen/nitrogen species and lack of the subtrates L-arginine and tetrahydrobiopterin, results in generation of •O_2_^−^. Thus, in addition to prevention of NO generation, the protective effect of L-NAME may be due to inhibition of •O_2_^−^ generated by un-coupled eNOS. There are other potential sources of reactive oxygen species such as xanthine oxidase and the mitochondrial respiratory chain. The role of NOX 2 and NOX 4 as a source of LTA-induced, ROS-dependent lung endothelial cell barrier dysfunction warrants further exploration.

### Conclusion

In summary, our data demonstrate that the TLR2 agonists LTA and PAM activate the TLR2 pathway which is associated with pulmonary endothelial barrier dysfunction. Moreover, ROS and RNS mediate the LTA mediated increase in lung endothelial permeability.

## Supporting Information

Figure S1 **TLR2 agonist activates the NFκB pathway in a dose-dependent manner.** *Top*: Representative Western blots of phosphorylated IκBα (Ser32/36) from PMEM treated for 1 hour with increasing concentrations of LTA or PAM. *Bottom*: Western blot band densities of blots represented in top panel. Values represent means ± SD, two samples per treatment.
(TIFF)

## References

[1] U.S. Renal Data System, USRDS Annual Data Report. (2009) Atlas of Chronic Kidney Disease and End-Stage Renal Disease in the United States, National Institutes of Health, National Institute of Diabetes and Digestive and Kidney Diseases, Bethesda, MD.

[2] Su CT, Hsueh PR, Wu VC, Wang CY, Hu FC, et al (2010) Maintenance haemodialysis and delayed administration of appropriate antibiotics increase 30-day mortality among patients with non-hospital-acquired meticillin-resistant Staphylococcus aureus bacteraemia. Int J Antimicrob Agents. 35(5): 511–2. 10.1016/j.ijantimicag.2009.12.016 20181464

[3] Chen H, Bai C, Wang X (2010) The value of the lipopolysaccharide-induced acute lung injury model in respiratory medicine. Expert Rev Respir Med. 4(6): 773–83. 10.1586/ers.10.71 21128752

[4] De Kimpe SJ, Hunter ML, Bryant CE, Thiemermann C, Vane JR (1995) Delayed circulatory failure due to the induction of nitric oxide synthase by lipoteichoic acid from Staphylococcus aureus in anaesthetized rats. British J of Pharmacol 214: 1317–1323. 10.1111/j.1476-5381.1995.tb13349.x PMC1510350 7542534

[5] Hoogerwer JJ, de Vos AF, Bresser P, van der Zee JS, Pater JM, et al (2008) Lung inflammation induced by lipotechoic acid or lipopolysaccharide in humans. Am. J Respir Crit Care Med 178: 34–41. 10.1164/rccm.200708-1261OC 18403723

[6] Santini M, Kutlesa M, Pangercic A, Barsic B (2010) The importance of pathogens in sepsis: Staphylococcus aureus story. Scand J Infect Dis. 42(3): 172–6. 10.3109/00365540903384141 19958239

[7] Morath S, von Aulock S, Hartung T (2005) Structure/function relationships of lipoteichoic acids. J Endotoxin Res. 11(6): 348–56. 10.1179/096805105X67328 16303090

[8] Leemans JC, Heikens M, van Kessel KPM, Florquin S, van der Poll T (2003) Lipoteichoic acid and peptidoglycan from Staphylococcus aureus synergistically induce neutrophil influx into the lungs of mice. Clinic and Diagnos Lab Immunol 10 (5): 950–953. 10.1128/CDLI.10.5.950-953.2003 PMC193888 12965932

[9] Xing J, Moldobaeva N, Birukova AA (2011) Atrial natriuretic peptide protects against Staphylococcus aureus-induced lung injury and endothelial barrier dysfunction. J Appl Physiol. 110(1): 213–24. 10.1152/japplphysiol.00284.2010 PMC3253004 21051573

[10] Kengatharan KM, De Kimpe S, Robson C, Foster SJ, Thiemermann C (1998) Mechanism of gram-positive shock: identification of peptidoglycan and lipoteichoic acid moieties essential in the induction of nitric oxide synthase, shock, and multiple organ failure. J Exp Med. Jul 20 188(2): 305–15. 10.1084/jem.188.2.305 PMC2212447 9670043

[11] Takeda K, Akira S (2005) Toll-like receptors in innate immunity. Internat Immunol 17(1): 1–14. 10.1093/intimm/dxh186 15585605

[12] Pietrocola G, Arciola CR, Rindi S, Di Poto A, Missineo A, et al (2011) Toll-like receptors (TLRs) in innate immune defense against Staphylococcus aureus. Int J Artif Organs. Sep 34(9): 799–810. 10.5301/ijao.5000030 22094559

[13] Volz T, Nega M, Buschmann J, Kaesler S, Guenova E, et al (2010) Natural Staphylococcus aureus-derived peptidoglycan fragments activate NOD2 and act as potent costimulators of the innate immune system exclusively in the presence of TLR signals. FASEB J. Oct 24(10): 4089–102. 10.1096/fj.09-151001 20522786

[14] Travassos LH, Girardin SE, Philpott DJ, Blanot D, Nahori MA, et al (2004) Toll-like receptor 2-dependent bacterial sensing does not occur via peptidoglycan recognition. EMBO Rep. Oct 5(10): 1000–6. 10.1038/sj.embor.7400248 PMC1299148 15359270

[15] Boueiz A, Hassoun PM (2009) Regulation of endothelial barrier function by reactive oxygen and nitrogen species. Microvasc Res. 77(1): 26–34. 10.1016/j.mvr.2008.10.005 19041330

[16] Lum H, Roebuck KA (2001) Oxidant stress and endothelial cell dysfunction. Am J Physiol Cell Physiol. 280;C719–C741. 10.1152/ajpcell.2001.280.4.C719 11245588

[17] Gertzberg N, Gurnani T, Neumann P, Forbes A, Jean-Louis N, et al (2007) Tumor necrosis factor-α causes barrier dysfunction mediated by tyrosine198 and tyrosine218 in ß-actin. Am J Physiol Lung Cell Mol Physiol 293: L1219–L1229. 17766589 10.1152/ajplung.00083.2007

[18] Bosma JW, Siegert CE, Peerbooms PG, Weijmer MC (2010) Reduction of biofilm formation with trisodium citrate in haemodialysis catheters: a randomized controlled trial. Nephrol Dial Transplant 25(4): 1213–7. 19948873 10.1093/ndt/gfp651

[19] Fournier B, Philpott DJ (2005) Recognition of Staphylococcus aureus by the innate immune system. Clin Microbiol Rev. 18(3): 521–40. 10.1128/CMR.18.3.521-540.2005 PMC1195972 16020688

[20] Lindner SC, Köhl U, Maier TJ, Steinhilber D, Sorg BL (2009) TLR2 ligands augment cPLA2alpha activity and lead to enhanced leukotriene release in human monocytes. J Leukoc Biol. 86(2): 389–99. 10.1189/jlb.1008591 19401382

[21] Powers JP, Li S, Jaen JC, Liu J, Walker NP, et al (2006) Discovery and initial SAR of inhibitors of interleukin-1 receptor-associated kinase-4. Bioorg Med Chem Lett 16(11): 2842–5. 16563752 10.1016/j.bmcl.2006.03.020

[22] Phelps DT, Ferro TJ, Higgins PH, Shankar R, Parker DM, et al (1995) Tumor necrosis factor-α induces the peroxynitrite-mediated depletion of lung endothelial glutathione via protein kinase C activation. Am. J. Physiol. 13: L–551-L559. 10.1152/ajplung.1995.269.4.L551 7485529

[23] Gertzberg N, Neumann P, Rizzo V, Johnson A (2003) NAD[P]H-oxidase mediates the endothelial barrier dysfunction induced by TNF-α. Am J Physiol 286: L37–L48. 10.1152/ajplung.00116.2003 12807699

[24] Patterson CE, Rhoades RA, Garcia JG (1992) Evans blue dye as a marker of albumin clearance in cultured endothelial monolayer and isolated lung. J Appl Physiol. 72: 865–873. 10.1152/jappl.1992.72.3.865 1568982

[25] Kawagoe T, Sato S, Matsushita K, Kato H, Matsui K, et al (2008) Sequential control of Toll-like receptor-dependent responses by IRAK1 and IRAK2. Nat Immunol. 9(6): 684–91. 10.1038/ni.1606 18438411

[26] Chen CA, Druhan LJ, Varadharaj S, Chen YR, Zweier JL (2008) Phosphorylation of endothelial nitric-oxide synthase regulates superoxide generation from the enzyme. J Biol Chem. 283(40): 27038–47. 10.1074/jbc.M802269200 PMC2556006 18622039

[27] Ozinsky A, Underhill DM, Fontenot JD, Hajjar AM, Smith KD, et al (2000) The repertoire for pattern recognition of pathogens by the innate immune system is defined by cooperation between toll-like receptors. Proc Natl Acad Sci U S A. Dec 5 97(25): 13766–71. 10.1073/pnas.250476497 PMC17650 11095740

[28] Agnihotri G, Crall BM, Lewis TC, Day TP, Balakrishna R, et al (2011) Structure-activity relationships in toll-like receptor 2-agonists leading to simplified monoacyl lipopeptides. J Med Chem. Dec 8 54(23): 8148–60. 10.1021/jm201071e PMC3228886 22007676

[29] Deininger S, Figueroa-Perez I, Sigel S, Stadelmaier A, Schmidt RR, et al (2007) Use of synthetic derivatives to determine the minimal active structure of cytokine-inducing lipoteichoic acid. Clin Vaccine Immunol. Dec 14(12): 1629–33. 10.1128/CVI.00007-07 PMC2168384 17928431

[30] Zivkovic A, Sharif O, Stich K, Doninger B, Biaggio M, et al (2011) TLR 2 and CD14 mediate innate immunity and lung inflammation to staphylococcal Panton-Valentine leukocidin in vivo. J Immunol. Feb 1 186(3): 1608–17. 10.4049/jimmunol.1001665 21178007

[31] Yipp BG, Andonegui G, Howlett CJ, Robbins SM, Hartung T, et al (2002) Profound differences in leukocyte-endothelial cell responses to lipopolysaccharide versus lipoteichoic acid. J Immunol. 168(9): 4650–8. 10.4049/jimmunol.168.9.4650 11971014

[32] Shin HS, Xu F, Bagchi A, Herrup E, Prakash A, et al (2011) Bacterial lipoprotein TLR2 agonists broadly modulate endothelial function and coagulation pathways in vitro and in vivo. J Immunol. 186(2): 1119–30. 10.4049/jimmunol.1001647 PMC3482611 21169547

[33] Neumann P, Gertzberg N, Vaughan E, Weisbrot J, Woodburn R, et al.. (2006) Peroxynitrite mediates TNF-alpha-induced endothelial barrier dysfunction and nitration of actin. Am J Physiol Lung Cell Mol Physiol. Apr;290(4): L674–L684, 2005. 10.1152/ajplung.00391.2005 16284212

[34] Tuanzhu HY, Hu L, Liu C, Lu JR, McMullen J, et al (2010) TLR2 ligands induce cardioprotection against ischaemia/reperfusion injury through a PI3K/Akt-dependent mechanism. Cardiovasc Res. 87: 694–703. 10.1093/cvr/cvq116 PMC2920812 20421349

[35] Kao SJ, Lei HC, Kuo CT, Chang MS, Chen BC, et al (2005) Lipoteichoic acid induces nuclear factor-kappaB activation and nitric oxide synthase expression via phosphatidylinositol 3-kinase, Akt, and p38 MAPK in RAW 264.7 macrophages. Immunology. 115(3): 366–74. 10.1111/j.1365-2567.2005.02160.x PMC1782163 15946254

[36] Li CJ, Elsasser TH, Kahl S (2009) AKT/eNOS signaling module functions as a potential feedback loop in the growth hormone signaling pathway. J Mol Signal. 25 4: 1. 10.1186/1750-2187-4-1 PMC2666727 19320971

[37] Kumar S, Sud N, Fonseca FV, Hou Y, Black SM (2010) Shear stress stimulates nitric oxide signaling in pulmonary arterial endothelial cells via a reduction in catalase activity: role of protein kinase C delta. Am J Physiol Lung Cell Mol Physiol. 298: L105–L116. 10.1152/ajplung.00290.2009 PMC2806197 19897742

[38] Kagan JC, Medzhitov R (2006) Phosphoinositide-mediated adaptor recruitment controls Toll-like receptor signaling. Cell 125(5): 943–55. 16751103 10.1016/j.cell.2006.03.047

[39] Laird MH, Rhee SH, Perkins DJ, Medvedev AE, Piao W, et al (2009) TLR4/MyD88/PI3K interactions regulate TLR4 signaling. J. Leukoc. Biol. 85: 966–977. 10.1189/jlb.1208763 PMC2698589 19289601

[40] Dunne A, Carpenter S, Brikos C, Gray P, Strelow A, et al (2010) IRAK1 and IRAK4 promote phosphorylation, ubiquitination, and degradation of MyD88 adaptor-like (Mal). J Biol Chem 285: 18276–18282. 20400509 10.1074/jbc.M109.098137 PMC2881752

[41] Tang CH, Hsu CJ, Yang WH, Fong YC (2010) Lipoteichoic acid enhances IL-6 production in human synovial fibroblasts via TLR2 receptor, PKC delta and c-Src dependent pathways. Biochem Pharmacol. 79(11): 1648–57. 10.1016/j.bcp.2010.01.025 20109438

[42] Tiwari RL, Singh V, Singh A, Barthwal MK (2011) IL-1R–Associated Kinase-1 Mediates Protein Kinase Cδ-Induced IL-1β Production in Monocytes. J. Immunol. 187: 2632–2645. 10.4049/jimmunol.1002526 21804018

[43] Xiong H, Zhu C, Li F, Hegazi R, He K (2004) Inhibition of Interleukin-12 p40 Transcription and NF-B Activation by Nitric Oxide in Murine Macrophages and Dendritic Cells. J Biol Chem 279: 10776–10783. 14679201 10.1074/jbc.M313416200

[44] Pendyala S, Usatyuk PV, Gorshkova IA, Garcia JG, Natarajan V (2009) Regulation of NADPH oxidase in vascular endothelium: the role of phospholipases, protein kinases, and cytoskeletal proteins. Antioxid Redox Signal. 11(4): 841–60. 10.1089/ars.2008.2231 PMC2850292 18828698

[45] Lee IT, Lee CW, Tung WH, Wang SW, Lin CC, et al.. (2010) Cooperation of TLR2 with MyD88, PI3K, and Rac1 in lipoteichoic acid-induced cPLA2/COX-2-dependent airway inflammatory responses. Am J Pathol. 176(4): 1671–84. Epub 2010 Feb 18. 10.2353/ajpath.2010.090714 PMC2843459 20167866

[46] Maitra U, Singh N, Gan L, Ringwood L, Li L (2009) IRAK-1 Contributes to Lipopolysaccharide-induced Reactive Oxygen Species Generation in Macrophages by Inducing NOX-1 Transcription and Rac1 Activation and Suppressing the Expression of Antioxidative Enzymes. J Biol Chem 284: 35403–35411. 19850916 10.1074/jbc.M109.059501 PMC2790969

[47] Park HS, Chun JN, Jung HY, Choi C, Bae YS (2006) Role of NADPH oxidase 4 in lipopolysaccharide-induced proinflammatory responses by human aortic endothelial cells. Cardiovasc Res. 72(3): 447–55. Epub 2006 Sep 23. 10.1016/j.cardiores.2006.09.012 17064675

[48] Wang LI, Lee SW, Chang CW, Lin CC, et al (2008) Lipoteichoic acid induces HO-1 expression via the TLR2/MyD88/c-Src/NADPH oxidase pathway and Nrf2 in human tracheal smooth muscle cells. J Immunol. 181(7): 5098–110. 10.4049/jimmunol.181.7.5098 18802114

[49] Chowdhury AK, Watkins T, Parinandi NL, Saatian B, Kleinberg ME (2005) Src-mediated tyrosine phosphorylation of p47phox in hyperoxia-induced activation of NADPH oxidase and generation of reactive oxygen species in lung endothelial cells. J Biol Chem 280(21): 20700–11. 15774483 10.1074/jbc.M411722200

