# Supplementary figures and images for: Lipoteichoic Acid from Staphylococcus aureus Induces Lung Endothelial Cell Barrier Dysfunction: Role of Reactive Oxygen and Nitrogen Species
Source: PLoS One. 2012 Nov 15;7(11):e49209. doi: 10.1371/journal.pone.0049209 (PMC3499573; doi:10.1371/journal.pone.0049209)

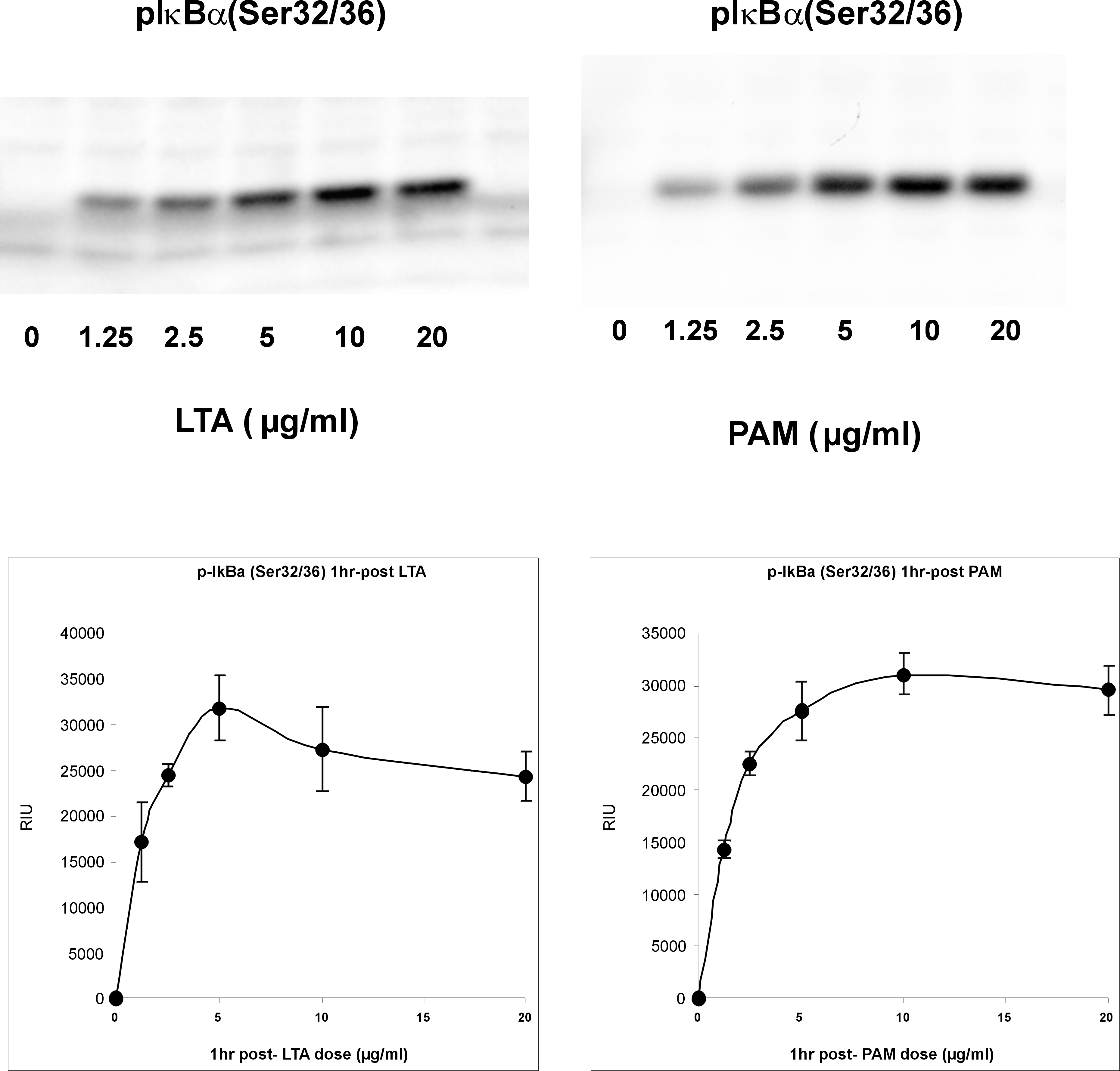

Supplement: Figure S1 — TLR2 agonist activates the NFκB pathway in a dose-dependent manner. Top: Representative Western blots of phosphorylated IκBα (Ser32/36) from PMEM treated for 1 hour with increasing concentrations of LTA or PAM. Bottom: Western blot band densities of blots represented in top panel. Values represent means ± SD, two samples per treatment. (TIFF) [file pone.0049209.s001.tiff]
